# Assessing compliance to reporting mandates in glioblastoma-related clinical trials

**DOI:** 10.1371/journal.pone.0323109

**Published:** 2025-05-16

**Authors:** Nicholas Kendall, Julian S. Rechberger, Abdelrahman M. Hamouda, Mark Cwajna, Sherief Ghozy, Kogulavadanan Arumaithurai, David F. Kallmes

**Affiliations:** 1 The University of South Dakota Sanford School of Medicine, Vermillion, South Dakota, United States of America; 2 Department of Neurologic Surgery, Mayo Clinic, Rochester, MinnesotaUnited States of America; 3 Saba University School of Medicine, Church Street, The Bottom, Dutch Caribbean,; 4 Department of Radiology, Mayo Clinic, Rochester, Minnesota, United States of America; 5 Department of Neurology, Mayo Clinic, Rochester, Minnesota, United States of America; Goethe University Hospital Frankfurt, GERMANY

## Abstract

**Introduction:**

Accurate and timely reporting of scientific knowledge is crucial to clinical research ethics. ClinicalTrials.gov allows researchers to register trials and report results to the public and scientific community. Despite FDA reporting mandates, compliance with the required 12-month window remains low. Given glioblastoma’s (GBM) aggressive nature, timely reporting is especially important for advancing research and benefiting patients. This study aimed to assess GBM trial reporting rates on ClinicalTrials.gov and identify factors related to non-compliance.

**Methods:**

We utilized a previously published algorithm to identify studies on ClinicalTrials.gov likely mandated to report. We obtained the titles, status, results, phases, funding type, intervention type, study design and type, location, and all available trial dates. Kaplan-Meier analysis evaluated reporting times, and Cox regression models identified factors associated with reporting within five years.

**Results:**

We identified 255 GBM-related trials likely mandated to report. 13% reported results within the 12-month deadline, while 82.7% reported within five years. Factors significantly associated with lower reporting rates at five years were biological interventions (HR 0.61, 95% CI: 0.37–1.00, p = 0.049), Phase 1–2 trials (HR 0.65, 95% CI: 0.46–0.91, p = 0.014), and studies with quadruple masking (HR 0.19, 95% CI: 0.04–0.93, p = 0.040).

**Conclusion:**

For GBM-related trials, noncompliance with reporting mandates remains a major issue. Reporting within 12 months was only 13%. No factors influenced reporting by 12 months, but multiple factors influenced five-year reporting. Further research is needed to understand these associations and create targeted incentives to increase transparency through timely reporting of GBM-related trials.

## Introduction

Since 2000, ClinicalTrials.gov has served as an online repository for past and current clinical trials. This site was initiated by the FDA as an attempt to promote transparency for both scientists and the public. Currently, the site is the largest database of clinical trials in the world and serves as an important tool for the organization and dissemination of scientific knowledge [1–3]. The reporting of study results, regardless of outcome, is crucial for scientific inquiry. Unfortunately, the issue of underreporting by scientists remains a problem for the scientific community [4]. Previous work has shown that despite the ethical importance of timely reporting, many clinical trials still delay the reporting of their results or withhold them indefinitely [5–7].

Seven years after the launch of the site, the FDA made an attempt to further incentivize reporting compliance by publishing the Food and Drug Administration Amendments Act (FDAAA 801, effective 2017). Included in this act was a mandation that researchers register all trials in the USA on the site, along with the reporting of their results to the site, regardless of publication status. The ability for the FDA to enforce this mandate did not appear until 2016, when they passed the Final Rule, which allowed for fines to be issued due to noncompliance. Under the Final Rule, the 12‐month reporting window begins on the trial’s primary completion date which is the day when final data for the primary outcome is collected. Sponsors must submit results to ClinicalTrials.gov within 12 months and the fines can be up to $10,000 a day against the sponsors of the trial [8]. However, compliance issues have persisted since then [7].

When viewed as a whole, understanding the factors influencing reporting remains challenging. However, by examining smaller cohorts of trials based on specific topics, researchers may gain clearer insights into which areas struggle or succeed with compliance and the underlying causes. Studies in oncology have shown higher reporting compliance than in some other fields, potentially due to public pressure and the visibility of cancer research [6,9]. Neurological trials, however, show inconsistent compliance, and neuro-oncology trials specifically exhibit significant reporting delays [10,11].

In this study, we aimed to evaluate the compliance of a critical field of neurological oncology. Glioblastoma (GBM) is a highly aggressive form of brain cancer that has an estimated mean survival rate of 15 months [12]. Clinical trials have been crucial for the improvement of GBM outcomes. Various clinical trials have shown incremental improvements in 2-year survival rates and an increased quality of life thanks to lessened therapy side effects [13]. For GBM patients, the stakes are high, making ethical research with rapid dissemination of results an essential standard. Researchers should adhere to regulatory guidelines in order to achieve continued advancements in treatment and care.

## Methods

### Data sources

To find studies listed on ClinicalTrials.gov that were likely to have been mandated to report, we followed a previously published algorithm for predicting highly likely applicable clinical trials (HLACTs). This algorithm was developed with input from the National Library of Medicine in 2015 by Anderson and colleagues [5]. We have recently utilized this algorithm in previously published studies on reporting rates [6].

**Fig 1:**
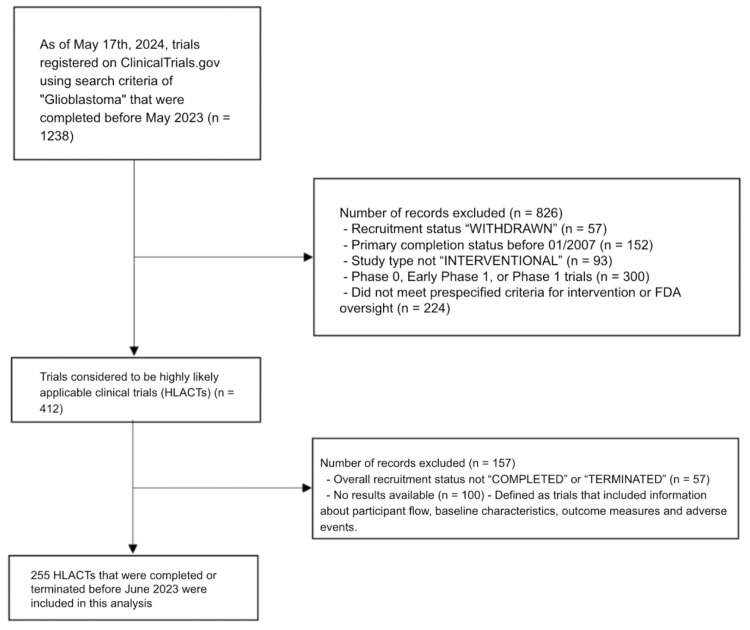
HLACTs Algorithm Visualization.

To utilize the algorithm, we first extracted data on trials from the site using the search criteria of trials completed before May 2023 under the search term “glioblastoma multiforme” This search also automatically included the following synonyms: glioblastoma, glioblastoma multiforme, glioblastomas, childhood high-grade cerebral astrocytoma, childhood glioblastoma, grade IV astrocytoma, childhood anaplastic astrocytoma, WHO Grade IV glioma, glioma glioblastoma multiforme, malignant glioblastoma, childhood glioblastoma multiforme, and pediatric glioblastoma multiforme. We completed our extraction on May 17th, 2024. We first filtered out all studies that were withdrawn, that had a primary completion status before January 2008 (the first year the original mandate expanded to require summaries of the results) and non-intervention studies. At this stage the algorithm also required the removal of studies that were phase 0, early phase 1 or phase 1 trials. Additionally, any trials that were unlikely to have FDA oversight were also removed. These exclusions leave a pool of trials that are considered HLACTs by this algorithm. Of those HLACTs, trials that were not completed or terminated were removed, as well as those with no available results (defined as trials that lacked information about participant flow, baseline characteristics, outcome measures and adverse events). All remaining HLACTs that ended before May 2023 were used for analysis in this study. This process is highlighted in Fig 1.

The clinicaltrials.gov site allows users to extract various aspects and categories regarding each study. For each study we obtained the titles, status, results, phases, type of funding, type of intervention, type of study, study design, location of the study and all available dates related to the trial. These dates allowed us to manually calculate how long it took for each study to report their results by calculating the months between the end of the trial and when they reported results to the site. We classified the funding sources as either industry funded, NIH funded or Other (which included universities and other government non-NIH funding such as the FDA, CDC, or the Department of Veterans Affairs) [14]. All study characteristics can be found in Table 1.

**Table 1:** Trial Characteristics.

Characteristic	All Trials (N = 255)	Trials with Results Reported by 12 months (N = 33)	Trials with Results Reported by 5 years (N = 211)
**Primary Purpose - no. (%)**			
Treatment	237 (92.94)	32 (96.97)	195 (92.42)
Diagnostic	11 (4.31)	1 (3.03)	10 (4.74)
Basic Science	2 (0.78)	0 (0.00)	2 (0.95)
Supportive Care	5 (1.96)	0 (0.00)	4 (1.90)
**Intervention Group - no. (%)**			
Drug	204 (80.00)	26 (78.79)	168 (79.62)
Radiation	19 (7.45)	5 (15.15)	18 (8.53)
Biological	26 (10.20)	2 (6.06)	19 (9.00)
Device	4 (1.57)	0 (0.00)	4 (1.90)
Genetic	2 (0.78)	0 (0.00)	2 (0.95)
**Phase - no. (%)**			
1-2	66 (25.88)	5 (15.15)	48 (22.75)
2	159 (62.35)	24 (72.73)	136 (64.46)
2-3	0 (0.00)	0 (0.00)	0 (0.00)
3	16 (6.27)	2 (6.06)	15 (7.11)
4	1 (0.39)	0 (0.00)	1 (0.47)
Not applicable	13 (5.10)	2 (6.06)	11 (5.21)
**Funding source - no. (%)**			
Industry	57 (22.35)	7 (21.21)	46 (21.80)
NIH	44 (17.25)	6 (18.18)	31 (14.69)
Other	154 (60.39)	20 (60.61)	134 (63.51)
**Primary completion year - no. (%)**			
2008	5 (1.96)	0 (0.00)	2 (0.95)
2009	13 (5.10)	1 (3.03)	9 (4.27)
2010	15 (5.88)	0 (0.00)	11 (5.21)
2011	15 (5.88)	0 (0.00)	13 (6.16)
2012	24 (9.41)	3 (9.09)	16 (7.58)
2013	18 (7.06)	1 (3.03)	15 (7.11)
2014	23 (9.02)	2 (6.06)	13 (6.16)
2015	19 (7.45)	6 (18.18)	17 (8.06)
2016	16 (6.27)	2 (6.06)	13 (6.16)
2017	12 (4.71)	1 (3.03)	9 (4.27)
2018	17 (6.67)	3 (9.09)	17 (8.06)
2019	22 (8.63)	4 (12.12)	21 (9.95)
2020	19 (7.45)	3 (9.09)	19 (9.00)
2021	13 (5.10)	5 (15.15)	13 (6.16)
2022	16 (6.27)	2 (6.06)	17 (8.06)
2023	6 (2.35)	0 (0.00)	6 (2.84)

### Statistical analysis

Descriptive statistics were computed, with Chi-square tests assessing significance for categorical variables. Kaplan-Meier survival analysis was performed to estimate the probability of reporting over time, with median reporting times and 95% confidence intervals calculated for subgroups. Statistical differences between these survival distributions were assessed with log-rank tests. To further identify independent predictors of reporting within specified time frames, descriptive statistics were used for reporting at 12 months due to scarce data variability, making it unsuitable to fit a multivariable regression model. For the 5-year mark, multivariable Cox proportional hazards models were applied to control for covariates like intervention type, trial phase, funding source, and completion status, with hazard ratios (HRs) and 95% confidence intervals calculated. All analyses were performed using R (R Core Team, 2024, version 4.4.2) and packages ‘finalfit’ and ‘survival,’ with significance defined as p < 0.05.

## Results

### Trial population

Our initial search yielded 1238 results. Of these trials, our algorithm identified 255 studies that were deemed HLACTs (the NCT identifier for each trial can be found in S1 table). Of the 255 trials, 33 reported within 12 months, 211 reported within 5 years, and 44 reported after the 5 year mark. Based on the data extracted from ClinicalTrials.gov, the vast majority (92.94%) of the studies had a “primary purpose” of treatment. 80% of the studies had a drug intervention group, followed by 10.2% of trials having a biological intervention group. 62.35% of all trials were phase 2 clinical trials, followed by 25.88% of trials being phase 1–2 trials (studies combining both phases in a single trial). The funding source for the trials was primarily listed as “other” (as previously defined) at 60.39% followed by 22.35% being industry funded and 17.25% were funded by the NIH. Our included trials spanned across 2008–2023 with 2012 having the highest number of studies completed in a year at 24 studies.

### Rates of reporting over time

**Fig 2:**
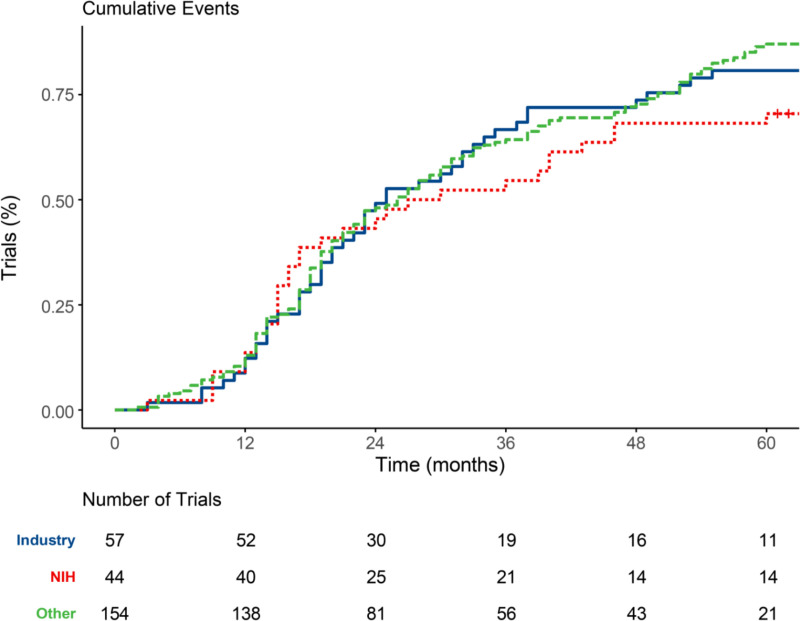
Kaplan-Meier Curves.

**Fig 3:**
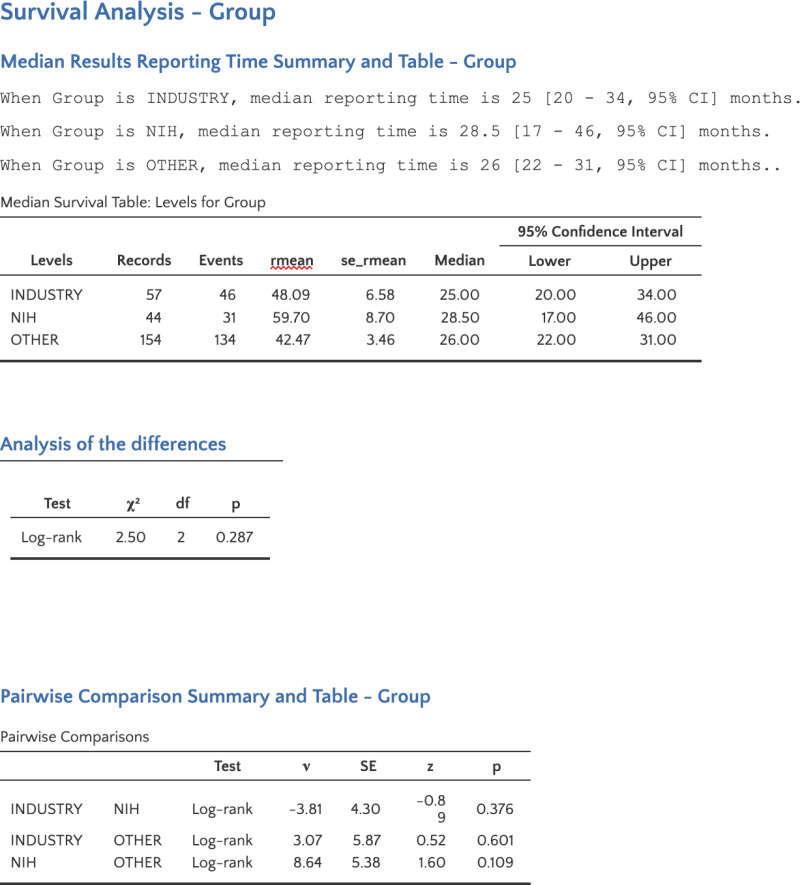
Detailed Kaplan-Meier Results.

**Fig 4:**
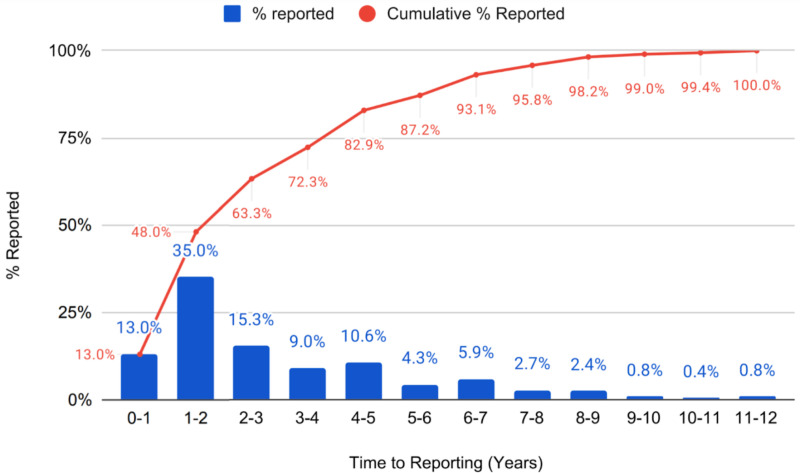
Study Reporting Compliance Over Time - Years.

**Fig 5:**
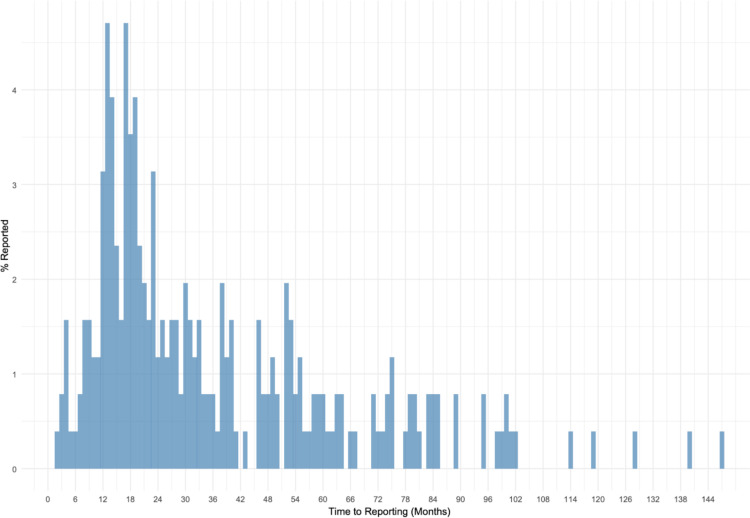
Study Reporting Compliance Over Time – Months.

The Kaplan-Meier analysis demonstrated no significant variation in median reporting times across the different funding source groups. Median reporting time was 25 months (95% CI: 20–34) for the Industry group, 28.5 months (95% CI: 17–46) for the NIH group, and 26 months (95% CI: 22–31) for the Other group. The log-rank test confirmed no statistically significant differences between the groups (χ² = 2.50, p = 0.287). Pairwise comparisons also indicated no significant differences between Industry and NIH (z = -0.89, p = 0.376), Industry and Other (z = 0.52, p = 0.601), and NIH and Other (z = 1.60, p = 0.109). These curves are visualized in Fig 2 and detailed Kaplan-Meier results are shown in Fig 3. In Fig 4, the reporting percentages are displayed in one-year increments and Fig 5 shows monthly increments.

### Reporting within 12 months and 5 years

The analyses of reporting times identified multiple statistically significant predictors for reporting within 5 years but none for 12-month reporting. For 5-year reporting, studies with biological interventions were significantly less likely to report compared to other types (HR 0.61, 95% CI: 0.37–1.00, p = 0.049). Phase 1–2 trials were less likely to report by 5 years when compared to Phase 2 trials (HR 0.65, 95% CI: 0.46–0.91, p = 0.014). Additionally, quadruple masking was associated with a reduced likelihood of reporting when compared to double masking (HR 0.19, 95% CI: 0.04–0.93, p = 0.040). Findings from each analysis are shown in Table 2 and Table 3. Additionally, there was no significant effect of the pre- vs. post-2017 period on 12-month reporting (10.1% pre-2017 compared to 16.8% post-2017, p = 0.132). However, a significant effect was observed on reporting within five years (73.6% pre-2017 to 95.3% post-2017, p < 0.001).

**Table 2:** Likelihood That Results of Clinical Trials Were Reported by 5 Years after the Primary Completion Date, According to Trial Characteristics.

Trial Characteristic	N (%)	Results Reported by 5 Yr
		*multivariable hazard ratio (95% CI)*
**Type of intervention**		
Drug	197 (81.4)	–
Biological	26 (10.7)	**0.61 (0.37–1.00, p = 0.049)**
Device	1 (0.4)	3.03 (0.41–22.58, p = 0.280)
Genetic	2 (0.8)	1.20 (0.29–5.00, p = 0.801)
Radiation	16 (6.6)	1.65 (0.93–2.93, p = 0.085)
**Trial Phase**		
2	159 (65.7)	–
1-2	66 (27.3)	**0.65 (0.46–0.91, p = 0.014)**
3	16 (6.6)	1.15 (0.48–2.73, p = 0.751)
4	1 (0.4)	5.57 (0.74–42.00, p = 0.095)
**Funding Source**		
NIH	43 (17.8)	–
Industry	57 (23.6)	0.82 (0.49–1.37, p = 0.440)
Other	142 (58.7)	0.98 (0.63–1.53, p = 0.936)
**Trial Terminated or Completed**		
Completed	184 (76.0)	–
Terminated	58 (24.0)	1.11 (0.78–1.59, p = 0.562)
**Use of Masking**		
Double	8 (3.3)	–
None	224 (92.6)	0.75 (0.34–1.67, p = 0.483)
Quadruple	4 (1.7)	**0.19 (0.04–0.93, p = 0.040)**
Triple	6 (2.5)	0.75 (0.23–2.49, p = 0.640)
**Primary Purpose of Study**		
Treatment	233 (96.3)	–
Basic Science	1 (0.4)	1.53 (0.20–11.86, p = 0.682)
Diagnostic	4 (1.7)	1.21 (0.44–3.33, p = 0.714)
Supportive Care	4 (1.7)	2.83 (0.79–10.17, p = 0.112)

¥ ^Multivariable hazard ratios were calculated by means of Cox regression. Regression models included the following covariates in addition to those listed: enrollment, year of study completion, and study duration.^

**Table 3:** Predictors of reporting by 12 months.

Variable		Reporting by 12 months		Total	P-value
**No**	**Yes**		
**Type of Intervention**	**Drug**	178 (80.2)	26 (78.8)	204 (80.0)	0.337
	**Biological**	24 (10.8)	2 (6.1)	26 (10.2)	
	**Device**	4 (1.8)	0 (0.0)	4 (1.6)	
	**Genetic**	2 (0.9)	0 (0.0)	2 (0.8)	
	**Radiation**	14 (6.3)	5 (15.2)	19 (7.5)	
**Trial Phase**	**2**	135 (64.0)	24 (77.4)	159 (65.7)	0.473
	**1-2**	61 (28.9)	5 (16.1)	66 (27.3)	
	**3**	14 (6.6)	2 (6.5)	16 (6.6)	
	**4**	1 (0.5)	0 (0.0)	1 (0.4)	
**Funding Source**	**NIH**	38 (17.1)	6 (18.2)	44 (17.3)	0.98
	**INDUSTRY**	50 (22.5)	7 (21.2)	57 (22.4)	
	**OTHER**	134 (60.4)	20 (60.6)	154 (60.4)	
**Trial Status**	**COMPLETED**	167 (75.2)	25 (75.8)	192 (75.3)	>0.99
	**TERMINATED**	55 (24.8)	8 (24.2)	63 (24.7)	
**Primary Purpose of Study**	**Treatment**	205 (92.3)	32 (97.0)	237 (92.9)	0.741
	**Basic Science**	2 (0.9)	0 (0.0)	2 (0.8)	
	**Diagnostic**	10 (4.5)	1 (3.0)	11 (4.3)	
	**Supportive Care**	5 (2.3)	0 (0.0)	5 (2.0)	
**Use of Masking**	**Double**	7 (3.2)	1 (3.0)	8 (3.1)	0.598
	**None**	203 (91.4)	32 (97.0)	235 (92.2)	
	**Quadruple**	4 (1.8)	0 (0.0)	4 (1.6)	
	**Triple**	8 (3.6)	0 (0.0)	8 (3.1)	
**Use of Randomized Assignment**	**Non-randomized**	57 (50.9)	9 (64.3)	66 (52.4)	0.508
	**Randomized**	55 (49.1)	5 (35.7)	60 (47.6)	

## Discussion

In this study, we aimed to understand the current state of compliance with reporting regulations for GBM-related clinical trials. Additionally, we explored study characteristics that may influence reporting times to help researchers and regulators better identify trials at risk of missing reporting deadlines and improve adherence to reporting protocols.

Our findings showed that, consistent with previous studies, non-compliance to the FDAs Final Rule remains a major issue in this area of research with only 13% of HLACTs reporting by the 12-month deadline and 82.7% reporting within 5 years. These rates are remarkably similar to the 2015 study that included 13,327 HLACTs across all types of clinical trials with 13.4% reporting within 12 months [5]. A 2021 oncology-trials study reported a similar finding, with a 10.6% reporting rate at 12 months [9]. Additionally, these rates are similar to previous research we have published on stroke related clinical trials which showed 16.5% reporting by 12 months and 89.6% reporting by 5 years [6]. As shown in Fig 4, 35% of studies reported their results after the 12-month deadline but before 24 months. Fig 5 breaks down reporting by month, showing that many studies narrowly missed the mandatory window. Together, these findings suggest that the 12-month mark was a target for many studies, but various factors may have contributed to delays.

Interestingly, there were no significant factors related to whether or not a study reported by 12 months. This contrasts with previous studies that have shown that industry sponsored studies had a higher compliance rate [5,7]. Additionally, our analysis showed no effect of the pre- vs. post-2017 timeframe, showing that the Final Rule has not influenced GBM trials. For future GBM-related clinical trials, this suggests a need for stronger incentives across all funding sources and study types to ensure compliance with reporting mandates. However, it is possible that no predictors were identified at the 12-month mark because the limited number of included studies reporting data for that time point prevented us from performing a regression analysis.

Our analysis of 5-year reporting found significant associations: trials with biological interventions, those in Phase 1–2, and studies employing quadruple masking had lower reporting rates. This trend may reflect the added complexity of these trials, potentially leading researchers to delay reporting, particularly when results are unfavorable. Anderson and colleagues additionally reported that rates vary by trial phase [5], while a similar study showed no such association^7^, indicating this is an important area for further investigation across all clinical trials, not just those related to GBM. Interestingly, among all included trials there was a low number of phase-3 studies (16/255). This could be because GBM treatments often fail early in development due to poor efficacy, toxicity, and the inherent heterogeneity of GBM, compounded by challenges such as a small patient population and high costs [15–17]. Furthermore, ethical concerns, regulatory hurdles, and a shift towards innovative, adaptive early-phase designs for novel therapies have limited the progression to large-scale phase-3 or phase-4 trials [18–20].

Additionally, the studies completed before 2017 had significantly lower reporting rates within five years. This suggests that, while the Final Rule may not impact compliance with the mandate, it has improved overall reporting timeliness. Given GBM’s high mortality and low survival rates, it is crucial that findings, favorable or not, are rapidly shared to advance treatment options. Likewise, due to the devastating nature of this diagnosis, it is essential for regulators to understand why researchers may not be compliant and to explore strategies to incentivize compliance. Future research could use surveys to investigate specific factors contributing to reporting delays and assess researchers’ familiarity with FDA reporting requirements. This approach could help determine whether noncompliance stems primarily from limited awareness of deadlines, resource constraints, or a combination of both. Based on the findings, targeted awareness campaigns or additional training for researchers on reporting timelines could be implemented. Integrating this qualitative data with our findings could enable more focused recommendations for regulators, addressing ongoing challenges in clinical trial reporting compliance and ultimately advancing treatment progress for serious conditions like GBM. Additionally, future comparisons between pediatric and adult trials could help further identify key areas for improvement outside of study characteristics.

Our work had several limitations. Similar to previous work, our algorithm for identifying HLACTs may not identify all possible trials that had mandated reporting, but rather is meant to estimate the compliance in a timely and reasonable manner. This means that there may be GBM-related studies that were not included or some that were incorrectly identified by the algorithm’s inclusion process. Additionally, researchers are able to request extensions from the FDA if they assess that they will be unable to meet the reporting deadline. This formalized process for approved delays was not assessed in our methodology.

## Conclusion

Our study highlights the persistent issue of non-compliance with mandatory reporting mandates in GBM-related clinical trials. Despite regulatory efforts, reporting rates remain low at the 12-month mark, regardless of study characteristics. However, our findings at the 5-year mark suggest associations that warrant further investigation to develop targeted incentives addressing the historical factors contributing to low compliance, not only in GBM trials but across all clinical trials. Prioritizing improvements in reporting mandates will enhance transparency and uphold ethical standards in clinical research in the USA.

## Supporting information

S1 Table List of NCT identifiers for all included clinical trials (DOCX)

## References

[1] Gresham G, Meinert JL, Gresham AG, Piantadosi S, Meinert CL. Update on the clinical trial landscape: analysis of ClinicalTrials.gov registration data, 2000-2020. Trials. 2022;23(1):858. doi: 10.1186/s13063-022-06569-2 36203212 PMC9540299

[2] Tse T, Fain KM, Zarin DA. How to avoid common problems when using ClinicalTrials.gov in research: 10 issues to consider. BMJ. 2018;361:k1452. doi: 10.1136/bmj.k1452 29802130 PMC5968400

[3] ClinicalTrials.gov. About ClinicalTrials.gov [Internet]. Bethesda (MD): National Library of Medicine (US); [cited 2024 Oct 26]. Available from: https://clinicaltrials.gov/about-site/about-ctg

[4] Stern JM, Simes RJ. Publication bias: evidence of delayed publication in a cohort study of clinical research projects. BMJ. 1997;315(7109):640–5. doi: 10.1136/bmj.315.7109.640 9310565 PMC2127436

[5] Anderson ML, Chiswell K, Peterson ED, Tasneem A, Topping J, Califf RM. Compliance with results reporting at ClinicalTrials.gov. N Engl J Med. 2015;372(11):1031–9. doi: 10.1056/NEJMsa1409364 25760355 PMC4508873

[6] Cwajna M, Hamouda A, Kendall N. Reporting compliance and factors influencing timeliness of stroke-related trial results on ClinicalTrials.gov. Transl Stroke Res. 2024;2024(Jun 3):1–6. 38831158 10.1007/s12975-024-01260-x

[7] DeVito NJ, Bacon S, Goldacre B. Compliance with legal requirement to report clinical trial results on ClinicalTrials.gov: a cohort study. Lancet. 2020;395(10221):361–9. doi: 10.1016/S0140-6736(19)33220-9 31958402

[8] Hyman P, McNamara PC. Food and Drug Administration Amendments Act of 2007 Summary and Analysis. Published October 17,2007. https://hpm.com/wp-content/uploads/2007/10/HPM-FDAAA-Summary-and-Analyss.pdf. Accessed 25Mar 2024

[9] Liu X, Zhang Y, Li WF, Vokes E, Sun Y, Le QT, Ma J. Evaluation of oncology trial results reporting over a 10-year period. JAMA network Open. 2021 May 3;4(5):e2110438doi: 10.1001/jamanetworkopen.2021.10438 34028549 PMC8144925

[10] Smith EJ, Naik A, Goel M, Wen PY, Lim M, Chang SM, Germano IM. Adult neuro-oncology trials in the United States over 5 decades: analysis of trials completion rate to guide the path forward. Neuro-Oncology Advances. 2024 Jan 1;6(1):vdad169. doi: 10.1093/noajnl/vdad169 38312230 PMC10838133

[11] Turner BE, Magnani CJ, Frolov A, Weeks BT, Steinberg JR, Huda N, Shah LM, Zuroff L, Gu BJ, Rasmussen H, Edwards JG. Neurology trial registrations on ClinicalTrials. gov between 2007 and 2018: A cross-sectional analysis of characteristics, early discontinuation, and results reporting. Journal of the Neurological Sciences. 2021;428:117579. 34332371 10.1016/j.jns.2021.117579

[12] Tamimi A, Juweid M. Epidemiology and outcome of glioblastoma. In: De Vleeschouwer S, editor. Glioblastoma. 29251870

[13] Angom RS, Nakka NMR, Bhattacharya S. Advances in Glioblastoma Therapy: An Update on Current Approaches. Brain Sci. 2023;13(11):1536. doi: 10.3390/brainsci13111536 38002496 PMC10669378

[14] ClinicalTrials.gov. Study Basics: Glossary. Bethesda (MD): National Library of Medicine (US); [cited 2024 Oct 26]. Available from: https://clinicaltrials.gov/study-basics/glossary. 2024. Accessed 2024 October 26

[15] Wu W, Klockow JL, Zhang M, Lafortune F, Chang E, Jin L, et al. Glioblastoma multiforme (GBM): An overview of current therapies and mechanisms of resistance. Pharmacol Res. 2021;171:105780. doi: 10.1016/j.phrs.2021.105780 34302977 PMC8384724

[16] Vanderbeek AM, Rahman R, Fell G, Ventz S, Chen T, Redd R, Parmigiani G, Cloughesy TF, Wen PY, Trippa L, Alexander BM. The clinical trials landscape for glioblastoma: is it adequate to develop new treatments?. Neuro-oncology. 2018 Jul 5;20(8):1034-43. doi: 10.1093/neuonc/noy027 29518210 PMC6280141

[17] Shergalis A, Bankhead III A, Luesakul U, Muangsin N, Neamati N. Current challenges and opportunities in treating glioblastoma. Pharmacological reviews. 2018 Jul 1;70(3):412-45. doi: 10.1124/pr.117.014944 29669750 PMC5907910

[18] Angom RS, Nakka NM, Bhattacharya S. Advances in glioblastoma therapy: an update on current approaches. Brain sciences. 2023 Oct 31;13(11):1536.doi: 10.3390/brainsci13111536 38002496 PMC10669378

[19] Singh K, Batich KA, Wen PY, Tan AC, Bagley SJ, Lim M, Platten M, Colman H, Ashley DM, Chang SM, Rahman R. Designing clinical trials for combination immunotherapy: a framework for glioblastoma. Clinical Cancer Research. 2022 Feb 15;28(4):585-93. 34561270 10.1158/1078-0432.CCR-21-2681 PMC9306329

[20] Rong L, Li N, Zhang Z. Emerging therapies for glioblastoma: current state and future directions. J Exp Clin Cancer Res. 2022;41(1):142. doi: 10.1186/s13046-022-02349-7 35428347 PMC9013078

