# Supplementary material for: Assessing compliance to reporting mandates in glioblastoma-related clinical trials
Source: PLoS One. 2025 May 16;20(5):e0323109. doi: 10.1371/journal.pone.0323109 (PMC12083813; doi:10.1371/journal.pone.0323109)
Supplement: S1 Table — (DOCX) [file pone.0323109.s001.docx]

**S1 Table**

| NCT00085566 | NCT00672243 | NCT00045110 | NCT00003476 | NCT00335764 | NCT00805961 | NCT01260467 | NCT01331291 | NCT02039778 | NCT01026493 | NCT02157103 | NCT02340156 | NCT01931098 | NCT02765165 | NCT02663271 | NCT03684811 |
| --- | --- | --- | --- | --- | --- | --- | --- | --- | --- | --- | --- | --- | --- | --- | --- |
| NCT00290771 | NCT00402116 | NCT00613028 | NCT00003457 | NCT00735436 | NCT00323115 | NCT00112736 | NCT01663012 | NCT02175745 | NCT05718466 | NCT02078648 | NCT01349660 | NCT02743078 | NCT02343549 | NCT02336165 | NCT03463265 |
| NCT00631137 | NCT00004146 | NCT00433381 | NCT00404248 | NCT00369590 | NCT00813943 | NCT00301418 | NCT01856933 | NCT00795665 | NCT01177397 | NCT00902577 | NCT01454596 | NCT02858895 | NCT02038218 | NCT01434602 | NCT03027388 |
| NCT00050986 | NCT00042991 | NCT00187486 | NCT00756106 | NCT01508117 | NCT00689221 | NCT00660543 | NCT01189240 | NCT01721577 | NCT01870726 | NCT01514201 | NCT01339052 | NCT00731731 | NCT01631552 | NCT03149003 | NCT03139916 |
| NCT00459381 | NCT00238303 | NCT00959946 | NCT00612339 | NCT00085254 | NCT00657267 | NCT00905060 | NCT01310868 | NCT01738646 | NCT01290263 | NCT02120287 | NCT02711137 | NCT01740258 | NCT02885324 | NCT01811498 | NCT03557372 |
| NCT00387894 | NCT00671970 | NCT00243022 | NCT01342757 | NCT01268566 | NCT00611325 | NCT01186406 | NCT01303835 | NCT01465347 | NCT01266031 | NCT00998010 | NCT01987830 | NCT00869401 | NCT02942264 | NCT03119064 | NCT02829723 |
| NCT00085540 | NCT00262730 | NCT01081223 | NCT00923117 | NCT01113463 | NCT00586508 | NCT01380782 | NCT01402063 | NCT01582152 | NCT02067156 | NCT01478854 | NCT02076152 | NCT00553150 | NCT02337491 | NCT01854554 | NCT01466686 |
| NCT00112866 | NCT00436436 | NCT00305864 | NCT00761280 | NCT00704288 | NCT01349036 | NCT01110876 | NCT01564914 | NCT01836549 | NCT01209442 | NCT02478164 | NCT01806675 | NCT02342379 | NCT02366728 | NCT01004874 | NCT03018288 |
| NCT00337207 | NCT00045708 | NCT00003475 | NCT00615927 | NCT00979017 | NCT01227434 | NCT01575275 | NCT01122901 | NCT01846871 | NCT01433991 | NCT00720356 | NCT02052648 | NCT01986348 | NCT03393000 | NCT03363659 | NCT01730950 |
| NCT00515086 | NCT00095940 | NCT00667394 | NCT00540722 | NCT01032200 | NCT00498927 | NCT00124657 | NCT01013285 | NCT00727506 | NCT00823797 | NCT03034135 | NCT02343406 | NCT03419403 | NCT01120639 | NCT02661282 | NCT00445965 |
| NCT00445588 | NCT00504660 | NCT00253448 | NCT00980343 | NCT00293423 | NCT01132547 | NCT00003458 | NCT01113398 | NCT00589875 | NCT01648348 | NCT02410577 | NCT02315534 | NCT01790503 | NCT02805179 | NCT02617589 | NCT03927222 |
| NCT00392171 | NCT00544817 | NCT00679354 | NCT00763750 | NCT00884741 | NCT00486603 | NCT01044966 | NCT00943826 | NCT02394665 | NCT00200161 | NCT01977677 | NCT00892177 | NCT02540161 | NCT00669669 | NCT03367715 | NCT02684058 |
| NCT00790452 | NCT00354913 | NCT00612430 | NCT00369785 | NCT00939991 | NCT01778530 | NCT00590681 | NCT01756352 | NCT00777153 | NCT01753713 | NCT00423735 | NCT01925573 | NCT02844439 | NCT03430791 | NCT02573324 | NCT01814813 |
| NCT00412542 | NCT00538850 | NCT00686725 | NCT00606008 | NCT00717197 | NCT01290692 | NCT00112502 | NCT02296476 | NCT01067469 | NCT00879437 | NCT01478321 | NCT02709889 | NCT03216499 | NCT01128218 | NCT01062425 | NCT03345095 |
| NCT00479765 | NCT00641706 | NCT01017653 | NCT00619112 | NCT00597402 | NCT00621686 | NCT01540513 | NCT00995007 | NCT00643097 | NCT00441142 | NCT01884740 | NCT02968940 | NCT02327078 | NCT03150862 | NCT01609790 | NCT03973918 |
| NCT00350727 | NCT00597493 | NCT01095094 | NCT00301873 | NCT00525525 | NCT00895180 | NCT00499473 | NCT01280552 | NCT00304031 | NCT01966809 | NCT01975701 | NCT01822275 | NCT02794883 | NCT02330562 | NCT01062399 |  |
